# Supplementary material for: Network determinants of relationship influence on HIV prevention decision-making among people in the social networks of women who have experienced incarceration in the US
Source: PLoS One. 2024 Oct 30;19(10):e0312584. doi: 10.1371/journal.pone.0312584 (PMC11524471; doi:10.1371/journal.pone.0312584)
Supplement: S1 Appendix — (PDF) [file pone.0312584.s001.pdf]

Interview Guide

Introduction

I'm recording now. I want to remind you that since we are recording, we're not going to use your name or anyone else's, just nicknames or initials, to help keep everything confidential. Even if you slip up and use someone's name, we will remove it from the interview. We will not be able to connect the things you tell us with your identity or anyone that you talk about during the interview.

Section 1: Network mapping (# minutes)

(Enumerate participants' sexual and drug-use partners, describe relationship duration and attributes, and qualitatively describe the extent to which and the ways in which other network members affect their HIV prevention decisions.)

Let's start by building an anonymous map of the relationships that have been important in your life in the past six months. You don't need to provide any names. As we build the map, I'll ask you some questions about those relationships.

<u>Friends</u>

Let's start by listing the friendships that have been important in the past six months. We'll just label them with initials or nicknames.

Great – now that we have this list I'm going to ask you some questions about each person. (To list).

<u>Family</u>

Now let's list the family members who have been important in your life in the past six months.

Okay – I'm going to ask you the same set of questions about each of these people. (To list).

<u>Romantic relationships and other sex partners</u>

Next will be romantic relationships. Let's list those from the past six months. And for each of these relationships... (to list)

Is there anyone else in the past six months that you have had sex with that you wouldn't say was a romantic relationship? We don't need to list those people if you wouldn't consider them important to you, but can you tell me how many people would be on that list?

<u>Drug partners</u>

Are there any people in the past six months that you used drugs with that you would consider to have an important relationship with you? Let's list them... And now I'll ask you a few questions about each. (To list)

Are there any other people that you have used drugs with in the past six months that you wouldn't say you had any kind of relationship with? How many people would be on that list?

Is there anyone else who we haven't talked about who you think should be on this list?

<u>*For each person listed:*</u>

1. How long has this person been an important part of your life?
2. How old is this person?
3. What is their race?
4. Does this person live in a city, town/suburb, or rural area?
5. Does this person use any kind of drugs? What kind? Do/Did you use any kind of drugs together?
6. Has this person ever been your sexual partner?
7. Is this person connected to any of the other people we've listed? How?
8. How does this person affect the things you do to prevent HIV, if at all?

Section 2: Perceptions of risks for HIV

1.0 Perceptions of participants' risks for HIV

1.1 What things make you more at risk for HIV?

1.2 What things make you less at risk for HIV?

2.0 Perceptions of participants' network risks for HIV

2.1 What about the people you have used drugs with? What makes them more at risk for HIV? Less at risk for HIV?

2.2How about the people you have had sex with? What makes them more at risk for HIV? Less at risk for HIV?

3.0 Perceptions of network influences on HIV risk

3.1 How do you think the people you use drugs with affect your risk of HIV?

3.2 What about the people you have sex with?

3.3 Are there other ways that the people you put on the network map affect your HIV risk?

In what ways do people in your network protect themselves from HIV?

How do you feel your social network perceives HIV prevention?

Section 3: ***PrEP as an HIV prevention intervention***

4.0 PrEP as an HIV prevention intervention

4.1 Have you ever heard of a medication to *prevent* HIV?

**If no: There is a daily medication, called PrEP, that can prevent you from getting HIV. I will provide you with some information at the end of the interview in case you'd like to find out more.

**If yes: Great, we are going to call that medication "PrEP" for the rest of the interview. I will provide you with some more information at the end of the interview in case you'd like to find out more.

4.2 Why might you decide to use PrEP?

4.3 Why might you decide to not use PrEP?

4.4 What kind of a reaction would you expect from a sex partner if you told them that you were using PrEP?

4.5 How might someone that you use drugs with react if you told them that you were using PrEP?

4.6 How might you feel talking with your network about HIV prevention?

4.6 How might you feel if a drug or sex partner asked you to consider using PrEP?

5.0 Barriers and facilitators to uptake of PrEP (motivation, financial constraints, relationship dynamics, and transportation)

5.1 What kinds of things would make you want to use PrEP?

5.2 What kinds of things would make it easier for you to take PrEP?

5.2 What things do you think might make it more difficult for you to take PrEP?

5.3 Is there anything else that you'd like to tell me about HIV prevention or PrEP?
