# Supplementary material for: Network determinants of relationship influence on HIV prevention decision-making among people in the social networks of women who have experienced incarceration in the US
Source: PLoS One. 2024 Oct 30;19(10):e0312584. doi: 10.1371/journal.pone.0312584 (PMC11524471; doi:10.1371/journal.pone.0312584)
Supplement: S1 Table — (PDF) [file pone.0312584.s002.pdf]

**Table S1. Comparison of AIC values for statistical models of relationship influence on PrEP and HIV prevention decision-making among participants recruited from the social networks of women who have experienced incarceration in the Southeastern US, 2020.**

| Models | AIC |
| --- | --- |
| Ego random intercepts | 301.9891 |
| Ego random intercepts and site fixed effects | 304.5450 |
| Ego fixed effects | 306.2322 |
| Baseline | 361.1242 |
