# Supplementary material for: Network determinants of relationship influence on HIV prevention decision-making among people in the social networks of women who have experienced incarceration in the US
Source: PLoS One. 2024 Oct 30;19(10):e0312584. doi: 10.1371/journal.pone.0312584 (PMC11524471; doi:10.1371/journal.pone.0312584)
Supplement: S2 Table — (PDF) [file pone.0312584.s003.pdf]

Table S2. Full model coefficients for the multi-level model of ego, alter, dyad, and network characteristics and relationship influence on PrEP and HIV prevention decision-making among participants recruited from the social networks of women who have experienced incarceration in the Southeastern US, 2020.

	**Dependent Variable:**<br>Influential relationship<br>*Coefficient (Standard error) (Odds ratio:)*
Site: site 3	−0.744 (1.017) (OR: 0.48)
Ego age	1.296* (0.706) (OR: 3.65)
Ego sex: male	−1.902 (1.450) (OR: 0.15)
Ego sexuality: non hetero	3.328*** (1.071) (OR: 27.87)
Ego race: white (reference: black)	−1.661 (1.192) (OR: 0.19)
Ego race: other (reference: black)	−0.023 (1.963) (OR: 0.98)
Alter has used drugs	−1.284* (0.768) (OR: 0.28)
Alter race: white (reference: black)	1.172 (0.891) (OR: 3.23)
Alter race: other (reference: black)	0.423 (1.040) (OR: 1.53)
Alter gender: Man	0.263 (0.436) (OR: 1.30)
Alter is or has been a drug partner	0.448 (0.739) (OR: 1.56)
Alter is or has been a sex partner	2.311*** (0.853) (OR: 10.09)
Relationship: parent (reference: friend)	0.767 (1.434) (OR: 2.15)
Relationship: other family (reference: friend)	−0.091 (1.025) (OR: 0.91)
Relationship: child or young family (reference: friend)	0.472 (1.080) (OR: 1.60)
Ego-alter absolute age difference	−0.366 (0.275) (OR: 0.69)
Ego and alter are the same race	−0.782 (0.901) (OR: 0.46)
Relationship duration	−0.185 (0.558) (OR: 0.83)
Proportion family	0.772 (0.501) (OR: 2.16)
Average relationship duration	−2.371*** (0.793) (OR: 0.09)
Average alter age	1.366** (0.546) (OR: 3.92)
Network contains at least one sexual partner	−1.565* (0.838) (OR: 0.21)
Network size	−0.139 (0.437) (OR: 0.87)
Constant	2.905* (1.694) (OR: 18.26)
Observations	216
Log Likelihood	-108.608
AIC	267.217
BIC	351.599

*Note: *p<0.1; **p<0.05; ***p<0.01*
