# Supplementary material for: Network determinants of relationship influence on HIV prevention decision-making among people in the social networks of women who have experienced incarceration in the US
Source: PLoS One. 2024 Oct 30;19(10):e0312584. doi: 10.1371/journal.pone.0312584 (PMC11524471; doi:10.1371/journal.pone.0312584)
Supplement: Attachment — Submitted filename: Response_to_reviewers-FINAL.docx [file pone.0312584.s004.docx]

Thank you very much for the opportunity to revise this manuscript. We have responded to the reviewers comments as below (italics) and believe that the manuscript is much improved for these changes.

Reviewer #1:

1. Thank you so much for the opportunity to review this manuscript which reports on a study investigating factors that influence decisions about HIV prevention. This is a thoughtfully designed study that builds on formative work in other contexts and from prior qualitative studies examining prevention decision making. This manuscript is also beautifully written. My major question is related to the analytic approach used and its appropriateness for network data. This concern and other observations are detailed below and shared with the intention of strengthening the presentation and contribution of this work.
   *Thank you. We have addressed the concerns below.*
2. I appreciated the content in the methods section that explained the interdependence in the data and the need for alternative approaches. However, I am left wondering whether the most appropriate analysis approach was used here. I’m curious why logistic regression was used instead of other analytic approaches that account for this nesting in networks (e.g. network autocorrelation models, mrQAP). The authors might need to revisit the analysis. If the current analysis is indeed the most appropriate for the data (compared to other autocorrelation approaches), then it would help to expand the paragraph in the methods section explaining why.
   *The reviewer correctly notes that other modeling approaches are available when dealing with data where observations are interdependent, such as autocorrelation models and MR-QAP. In this case, we were interested in the way that local network composition and structure affect the perceptions of ego. Our theory indicated that these associations might be at the network-, dyad-, relationship- and individual-level. MR-QAP is typically used when testing association between two or more dyadic features (e.g., perceived influence, closeness, time known). It would not allow for the inclusion of structural or personal variables without first transforming them into their dyadic alternatives. Network autocorrelation models are useful when there is a concern for cross-ego autocorrelation (i.e. egos within a bounded sociocentric network). In the case of ego-network analysis, egos are assumed to be independent. A hierarchical probability model is the standard supported by reviews of ego-network analysis. It combines the two benefits described above: (1) it allows for the inclusion of associations at all three levels (structural, dyadic and individual) (2) it breaks up the variance between and within ego, where we have concerns for correlation. While a linear probability model produces coefficients that are easier to interpret, we think that logit fits the functional form better and produces more conservative estimates. Following the reviewer’s suggestion, we expand on this point in the methods section as follows:

   This modeling approach has two main benefits over other popular alternative network models that account for the interdependence of observations in a network setting – quadradic assignment procedures (QAP) and network autocorrelation models. QAP are a non-parametric approach that tests significance between two or more dyad or relationship variables (e.g, shared race, closeness, time known). QAP does not allow for simultaneous tests of associations between network, dyad, relationship and individual-level variables. Network autocorrelation models are useful when there is a concern for cross-ego autocorrelation (i.e. egos within a bounded sociocentric network). In our case, the assumption that each network represents the independent, local view of an ego is more reasonable because the data were collected as ego networks rather than from within a single larger network, and because many of the egos were recruited from different sites, the networks include multiple types of connections (e.g., family, friends), and most recruiters only recruited 1-2 participants. The hierarchical logistic model selected for this study is supported by current methodological reviews of ego-network analysis (see, for example, Perry, Pescosolido, & Borgatti, 2018) because it enables both a contrast of variables across multiple levels, and a contrast of within and between ego variation.*
3. Regarding the analysis, was the amount of time since release from incarceration assessed? The composition of ego networks, strength of ties, and influence is likely to change over time after release and might be something to control for in analysis.
   *We did ask participants (egos) about the timing of their last incarceration. We have added the following sentence to the results:

   Of those with a history of incarceration, 12 (41.2%) were within the past year, 5 (17.2%) were during the prior year, and 19 (65.5%) were before the prior year.

   We did not include the amount of time since release from incarceration in the models since several participants did not have a history of incarceration and others had experienced multiple episodes of incarceration in the past but we did not systematically gather data on these other exposures.*
4. Similarly, did the analysis control for recruiter since participants were recruited from their own social network?
   *We noted that many recruiters only recruited 1-2 participants and so we did not include this in the model. We did test to see whether adding recruiter improved the fit of the model, but it did not. We have added the following sentence to the manuscript:

   Most recruiters only referred 1-2 participants.

   We also added the following explanation about the analysis:

   The final model included the ego characteristics of age, race, gender, and recruitment site. History of incarceration was not included as there were very few individuals who had not been incarcerated and specific recruiter was not included as most recruiters only recruited 1-2 participants. It included alter characteristics of age, race, pronouns, and substance use, dyad characteristics of race concordance and age difference, relationship characteristics of prior substance use together, sexual relationship, relationship type (friend, parent/guardian, child, other family), and relationship duration. It also included network characteristics of network size, average age, presence of a sexual relationship (yes/no), proportion of family in the network, average relationship duration.*
5. Include the interview guide so others can understand exactly what was asked and where investigators abstracted information.
   *We have added the interview guide as Appendix A.*
6. Were participants asked about relationships among alters in their social network? I’m wondering how this information was gathered to allow for ego network density calculations.
   *Yes. For each individual who was enumerated, the participant was asked “Is this person connected to any of the other people we’ve listed? How?” We have added the following to the measures portion of the methods section:

   Network density was defined as the number of existing relationships within the network out of all possible relationships within the network. Relationships between alters were assessed during the qualitative interview by asking “Is this person connected to any of the other people we’ve listed? How?”*
7. The language of influence can get a little tricky and I appreciate how this study focused on ego’s reports of alters influence (yes/no) rather than the valence of the influence (positive or negative). That said, the analysis generates results that suggest that a factor is positively associated (meaning exerts more influence, not necessarily positive or negative) or negatively associated (meaning exerts less influence) - if I’m reading this right. The authors were very careful with how they wrote about this and it might be helpful to call out the emphasis on influence (rather than valence) in the discussion section.
   *This is an excellent point. We have added the following to the methods section:

   Notably, we did not code for the valence of the influence (i.e., whether the relationship was supportive of HIV prevention activities versus discouraging of HIV prevention activities.)*

   *We have also added the following to the discussion section:

   Finally, we coded for the presence or absence of influence around HIV prevention in relationships, not whether that influence was positive or negative toward health promoting activities. Our qualitative analyses of these interviews do suggest that many egos understood the question about influence to be asking in particular about positive influence in support of HIV prevention; these egos responded with examples of partners, friends, and family supporting them to engage in behaviors or make decisions that would help to avoid HIV acquisition.*
8. As authors refine this manuscript it might help to keep the network-naive reader in mind by keeping language consistent (e.g. tie or dyad; ego networks) throughout the paper. Likewise, the specific dyad-level and network measures generated (and how) might be presented in a complementary table.
   *We have revised the language throughout for consistency. Thank you for this suggestion. We have also revised the text of the methods as follows to describe how the measures were generated, obviating the need for another table.

   Interview responses were aggregated to construct relevant structural features of participants' social networks at three levels, dyad, relationship, and network.*

*Dyad Characteristics. Dyad-level variables compare characteristics of our participants and their network alters (e.g., whether the dyad shares the same race). Dyad-level variables were constructed as binary variables indicating similarity or dissimilarity on each of the individual characteristics of age and sex. These were coded as a match or not between the ego and each of the alters’ characteristics.*

*Relationship Characteristics. Relationship-level variables explore the qualities of the connection in a given dyad (e.g., how long the dyad has known each other). Relationship-level measures of sexual relationship history, experience with sharing drugs, duration of relationship and type of relationship (i.e., family or friend) were abstracted from participant interviews. Sexual relationship and history of substance use together were binary variables indicating the presence or absence of each type of relationship. Types of relationships were first coded as friendships or family relationships. Family relationships were then further parsed into parental/guardian relationships if the duration of the relationship was equal to the age of the ego; as a child or younger sibling relationship if the alter was under 18 years old; or as another family member if neither of the previous categories were true. Relationship duration was abstracted in any reported unit of time and transformed to years for analysis.*

*Network Characteristics. Network-level variables encompass the ego’s entire ego-network such as overall composition (e.g., proportion of alters who are family members) or structure (e.g., network size, density).*

*Network-level measures were aggregated to reflect the composition of the ego-network as a whole. The proportion of network alters who were family members or friends was defined as number of family or friend alters over the total number of alters. The average relationship duration and average alter age in the network were calculated as means with standard deviations. We also used a binary indicator of the presence or absence of a sexual partner in the network as a whole. Network size was a count of all of the alters in a given ego-network. Network density was defined as the number of existing relationships within the ego-network out of all possible relationships within the ego-network. Relationships between alters were assessed during the qualitative interview by asking “Is this person connected to any of the other people we’ve listed? How?” Any affirmative response was coded as an existing relationship.*

9. Out of curiosity, how does excel generate network graphs?!
   *We used the NodeXL plugin in Excel to generate the network graphs. We have added the following sentence to the methods:*

*To generate the network graphs, we utilized the NodeXL plugin for Microsoft Excel, which allows users to input data from quantitative or qualitative sources and create network visualizations.*

10. There are a very small number (n=3) of participants who reported Asian or Latinx race/ethnicity and there might be privacy concerns (risk of deduced identity). The authors might consider rephrasing how they describe these participants.
    *Thank you for this excellent point. We have revised this sentence to state that “…the additional participants reporting another racial/ethnic identity (n=3, 9%);”*

Reviewer #2

11. This article describes an interesting mixed-methods study examining the influence of social networks at individual-, dyad-, relationship-, and network-levels on decision-making around PrEP use for 32 women who have experienced incarceration. The findings have important clinical, policy, and research implications. Overall, this study is well executed, used appropriate methodology, and is well-written. Thus, I am recommending acceptance with minor revisions with the following points requiring clarification.
    *Thank you very much. We have made revisions in response to these points below.*
12. There were some minor typos and grammar issues that did not significantly detract from the meaning of the article, but could use another round of proofreading.
    *We have reviewed the manuscript again for any grammar and style issues and have corrected those that we identified.*
13. The formatting of tables and figures need to be aligned with APA style (if this is related to how the manuscript is uploaded to the site, please ignore)
    *We have revised the tables and figures to be consistent with the PLOS ONE guidance (*[*https://journals.plos.org/plosone/s/tables*](https://journals.plos.org/plosone/s/tables)*).*
14. The study is inconsistently described throughout as qualitative, quantitative, and mixed-methods. I suggest that the authors describe it as mixed-methods throughout to reduce confusion.
    *Thank you very much for pointing this out. We have revised throughout with particular attention to removing “qualitative” or “quantitative” as modifiers when using “network analysis” or “interview” would be clearer.*

    Introduction:
15. Please add details from the cited references on the underlying causes of HIV acquisition for incarcerated women to prevent simply stigmatizing the population as “incarcerated women = higher risk of getting HIV” as it reads now.
    *We have revised this sentence to read as follows in order to convey the complexity of HIV acquisition in the community after incarceration:

    The experience of incarceration for cisgender women in the US is one of a tangled web of interconnected and interrelated biological and social factors, also called a syndemic, that produces disparities in HIV acquisition after return to the community (Kelly, 2014; Koblin et al., 2015; Singer et al., 2006). The syndemic is driven by shared risk factors for HIV and incarceration (i.e., substance use and sex exchange), social and structural marginalization based on poverty, racism, and substance use, and the disruptive effects of women’s incarceration on their social, sexual, and support networks (Fogel et al., 2014; Herbst et al., 2016; Knittel et al., 2021).*
16. Similarly, why is PrEP use low in this population? Why is it different from the general women's population?
    *We have added the following sentence to the first paragraph of the introduction:

    Many of the hesitations and barriers to PrEP initiation among women with incarceration histories and women recruited for research based on other experiences are similar, including low perceived need for PrEP, stigma, cost, and medical distrust (Auerbach, Kinsky, Brown, & Charles, 2015; Knittel, Ferguson, Jackson, & Adimora, 2022; Knittel et al., 2023). These similarities may result from the very high prevalence of incarceration experience among all women at heightened risk for HIV acquisition (Knittel et al., 2021).*
17. Is there research comparing the social and sexual networks of incarcerated and non-incarcerated cisgender women around decision-making and/or HIV prevention? If so, what are the differences, similarities? If not, it would be good to note this.
    *We have added the following sentence to convey this research:

     Research on women’s incarceration and their sexual networks shows that relationship disruption due to incarceration may produce concurrent partnerships, or partnerships that overlap in time, and new partnerships following incarceration (Khan et al., 2008; Knittel, Lambdin, Comfort, Kral, & Lorvick, 2019; Knittel et al., 2020).*
18. What hypotheses did you have to guide your analyses? These are indicated in the third paragraph, but not clearly stated.
    *We have added the following sentence to the last paragraph of the introduction:

    We hypothesized that sexual partnerships and family relationships would be influential, while relationships that included substance use would not be influential.*

    Methods:
19. Overall, the descriptions of variable operations, the selection of methodology, and the analytic plan are well-written. They are very clear and easy to follow, even for readers who are not from the same field. The blinded interviews were a good choice to prevent bias from knowing participants’ background information.
    *Thank you.*
20. Although the sample size was small, it may still be worthwhile to display the number of participants who were awarded or not awarded PrEP as a characteristic if recorded. There might be differences between these two groups when asked to identify the influence of their networks on HIV prevention.
    *We did not ask individuals whether they had received PrEP.*
21. Since you are measuring density, I’m assuming that you assessed whether dyads of alters had ties to each other as well as the ego-alter dyad. Please clarify this in the description of the network characteristics measurement.
    *Yes. For each individual who was enumerated, the participant was asked “Is this person connected to any of the other people we’ve listed? How?” We have added the following to the measures portion of the methods section:

    Network density was defined as the number of existing relationships within the network out of all possible relationships within the network. Relationships between alters were assessed during the qualitative interview by asking “Is this person connected to any of the other people we’ve listed? How?”*
22. Were characteristics such as socioeconomic status and health insurance coverage collected in the dataset ,as these may be barrier to access PrEP?
    *We did not assess these systematically. The qualitative interview guide is now included as Appendix A in order to answer reader/reviewer questions about what was included. We have previously published a qualitative analysis of these interviews that does highlight socioeconomic status and health insurance coverage as potential barriers. Since the participants were enumerating network alters, we did not think they would be able to reliably report SES or health insurance coverage.*
23. If using pronouns to identify 'sex', it would be better to use 'gender' instead of 'sex' as the name of the category.
    *We have made the recommended change throughout.*
24. “Substance use was phrased as “drug use” in the interview, and alcohol was included if the ego considered the alter’s use of alcohol to fall into the category of “drug use” for that alter.” ◊ Did the interviewers ensure that the egos considered alcohol use as drug use? If not, this could lead to some bias in the data analysis.
    *When we conducted the interviews, we did not specify that alcohol should be considered drug use. As a result, when looking at the text of the interviews, we suspect that problematic alcohol use, but probably not social drinking that did not meet criteria for alcohol use disorder treatment was included. We have added the following clarification to the methods section.

    We did not specifically prompt the participants to consider alcohol use and, based on review of the interview transcripts, alcohol was discussed explicitly when an alter was in treatment for alcohol use disorder or if the alcohol use was considered problematic for the alter by the ego.

    It is possible that this led to bias in the data. We have added the following to the limitations:*

    *We also assessed alter substance use via the report of the ego, so it is possible that some substance use was unknown to the ego and that alcohol use, in particular, may not have been included as substance use if the ego did not perceive it to rise to the level of problematic use for the alter.*
25. The use of terms like “higher level egos” and “lower level alters” creates confusion without clearer explanation. I am interpreting this as relating to the nesting / hierarchical structure for analysis. If this is the case, I’d suggest rewording this to be more explicit that you are describing the nesting structure.
    *We have revised this section to describe the analysis more clearly. The sentence that previously used the higher/lower level terms now reads as follows:

    Personal networks are naturally clustered at two levels: the participant (“ego”) who is observed directly, and their network members with their associated connections (“alters” and “relationships”).

    The description of the hierarchical models now reads as follows:*

    *However, multilevel modeling (van Duijn, van Busschbach, & Snijders, 1999), and more specifically hierarchal multilevel models (Goldstein, 2011; McCarty, Lubbers, Vacca, & Molina, 2019) account for the nested structure of personal networks (Vacca, 2018), where the alters can be specified as one level, nested within the higher level of the egos.*
26. You specify that you are focusing on cisgender women as the egos in this study, but 12.5% of the sample reported male sex. Why were they included in the sample? What are the potential implications or complications of their inclusion?
    *Thank you for pointing this out. We have revised the descriptions of the focus of the study to be clear that we recruited the egos from the networks of cisgender women. We have revised the footnote as follows:

    The authors recognize that both cisgender and transgender women who experience incarceration bear a disproportionate burden of HIV risk in the US. Because of the different experiences of these groups of women depending on the facilities in which they were incarcerated (i.e., “men’s” facilities versus “women’s” facilities, with sex at intake usually assigned based on sex assigned at birth), it is important to consider them separately. This work focuses on individuals recruited by cisgender women from their networks.*

    *We have revised the last paragraph of the introduction as follows:

    Given the themes related to relationships and networks that emerged in our prior work, we sought in this study to conduct a network analysis of networks reported by individuals recruited by cisgender women who had incarceration histories. We aimed to identify determinants of influence on HIV prevention decisions, including PrEP. We hypothesized that sexual partnerships and family relationships would be influential, while relationships that included substance use would not be influential.

    We have revised the description of the participant recruitment as follows:*

    *We did not specify any sex/gender limitations on the individuals who were referred.*

    Results:
27. The results section is clearly written and the tables and figures are strong additions and aid in the interpretation of the results.
    *Thank you.*

    Discussion:
28. “Taken together, these findings have important implications for the implementation of network interventions among women who have experienced incarceration and their social and sexual networks.” Please specify here that the network interventions would be focused on HIV prevention behaviors.
    *We have revised this paragraph as recommended to read as follows:

    Taken together, these findings nonetheless have important implications for the implementation of network interventions focused on HIV prevention among women who have experienced incarceration and their social and sexual networks. For change agent and induction interventions, our results suggest that effectiveness of individuals selected to promote HIV-related behavior change or the particular peer-to-peer interactions that are stimulated may vary substantially based on the specifics of those relationships and shared experiences (e.g., shared experiences of incarceration versus shared history of substance use). For segmentation interventions, targeting groups of individuals to change behavior together, such as adopting PrEP or increasing condom use, without including sexual partners may be more difficult based on our finding that sexual relationships may draw influence away from the rest of the network. The potential for leveraging sexual partnerships directly is complicated by prior findings that partners may be untrustworthy and in the most extreme circumstances may be physically or emotionally abusive (E. F. Dauria et al., 2021; Knittel et al., 2022; Knittel et al., 2023), but where possible, these highly influential relationships may be important conduits for behavior change. This research did not address the potential for transmission of dis- or mis-information about HIV prevention within networks, although this is likely to be another important consideration going forward. Overall, these findings support using nuanced network approaches to behavior change for HIV prevention that respect and leverage the diversity of relationships that comprise the social networks of women who have experienced incarceration.*
29. You identify a number of important practice / intervention implications. In addition to these, what are the possible public health policy implications? What future research do you suggest that could build on your findings?
    *We have expanded these last paragraphs to now include a paragraph each on future research and health policy implications. They are as follows:*

    *The results of this study also raise important questions for future research. Beyond ego-networks, efforts toward analyses of longitudinal network data, complete networks across communities or organizations, and information networks that include online alters would all provide important additional perspectives on the ways that women who have experienced incarceration and individuals in their networks make decisions about HIV prevention. Larger network studies building on these findings could measure HIV-related knowledge, risk perception, and prevention behaviors across networks and over time to empirically understand diffusion of information and behaviors across networks that include cisgender women with histories of incarceration. Additionally, we did not address the potential for transmission of dis- or mis-information about HIV prevention within networks, although this is likely to be an important determinant of HIV prevention behaviors.*

*Taken together, these findings nonetheless have important implications for the implementation of network interventions focused on HIV prevention among women who have experienced incarceration and their social and sexual networks. For change agent and induction interventions, our results suggest that effectiveness of individuals selected to promote HIV-related behavior change or the particular peer-to-peer interactions that are stimulated may vary substantially based on the specifics of those relationships and shared experiences (e.g., shared experiences of incarceration versus shared history of substance use). For segmentation interventions, targeting groups of individuals to change behavior together, such as adopting PrEP or increasing condom use, without including sexual partners may be more difficult based on our finding that sexual relationships may draw influence away from the rest of the network. The potential for leveraging sexual partnerships directly is complicated by prior findings that partners may be untrustworthy and in the most extreme circumstances may be physically or emotionally abusive (E. F. Dauria et al., 2021; Knittel et al., 2022; Knittel et al., 2023), but where possible, these highly influential relationships may be important conduits for behavior change.*

*There are also lessons to take from this study for policy approaches to eliminate HIV acquisition among women who have experienced incarceration. Policies aimed at addressing structural-level barriers to HIV prevention, such as improving insurance coverage and building clinical capacity for PrEP (Pinto, Berringer, Melendez, & Mmeje, 2018) will also need to contend with network dynamics. One way this could be operationalized would be to prioritize insurance coverage strategies that are not determined or affected by marital status either via spousal employment or income. Another would be to emphasize capacity for PrEP provision in the places that people already go to receive other services from trusted providers, such as primary care offices and sexual and reproductive health clinics (Pleuhs, Quinn, Walsh, Petroll, & John, 2020), in order to provide another influential relationship that might counterbalance negative HIV prevention influences in individual networks.*

*Overall, these findings support using nuanced network approaches to behavior change for HIV prevention that respect and leverage the diversity of relationships that comprise the social networks of women who have experienced incarceration.*

Tables / figures / supplemental material:

30. Table S2: Suggest adding heading labels to indicate the coefficient, CI, SE, and OR for the table. This will help readers grasp the results more easily.
    *Thank you, we have made this change.*
